# Infantile Sinonasal Tract Myxomas with Orbital Involvement: Presentation of Two Cases and Comprehensive Literature Review

**DOI:** 10.3390/jcm13226818

**Published:** 2024-11-13

**Authors:** Mason Jenner Burns, Nicole S. Graf, Megan Hobson, Ali Moghimi, Krishna Tumuluri

**Affiliations:** 1 Faculty of Medicine and Health, University of Sydney, Sydney, NSW 2006, Australia; 2 Ophthalmology Department, The Children’s Hospital at Westmead, Sydney, NSW 2145, Australia; 3 Department of Histopathology, The Children’s Hospital at Westmead, Sydney, NSW 2145, Australia; 4 Westmead Clinical School, Discipline of Clinical Ophthalmology and Eye Health, University of Sydney, Sydney, NSW 2145, Australia; 5 Department of Ophthalmology, Faculty of Medicine and Health Sciences, Macquarie University, Sydney, NSW 2019, Australia; 6 Save Sight Institute, Central Clinical School, Discipline of Clinical Ophthalmology and Eye Health, University of Sydney, Sydney, NSW 2006, Australia

**Keywords:** sinonasal tract myxoma, pediatric, cranial fasciitis, WNT/β-catenin, orbital tumor

## Abstract

**Objective:** Our aim is to present two cases of infantile sinonasal tract myxoma with orbital involvement and conduct a comprehensive literature review of the topic. We aim to provide a summary of the presentation of infantile sinonasal tract myxomas to effectively aid clinicians in considering this rare entity as a potential diagnosis. **Methods:** We present a case series and a retrospective review of the published literature in the English language. A search was conducted between 1945 and 2023 on sinonasal myxoma. Cases of infantile (<3 years) sinonasal myxoma in the literature were reported as well as two cases of our own. Data was collected from each of the identified articles on the age of presentation, sex, initial presentation, tumor location, imaging, pathology, and treatment. **Results:** Forty-eight cases of sinonasal myxoma in children <3 years of age were identified. These cases had a slight male preponderance (F:M, 1:1.39) and an average age of diagnosis of sixteen months. Of the cases included, the majority involved the maxilla and maxillary sinuses (83.3%) and commonly presented with a painless slow-growing mass in the region of the tumor. Characteristic CT findings are homogenous solid masses with heterogeneous contrast enhancement. On MRI, these lesions appear hypointense on T1 with highly variable contrast enhancement and hyperintense on T2. **Conclusions:** We present two cases of infantile sinonasal myxoma with secondary orbital involvement followed by a major review. Treatment of these cases with surgical excision (confirmed clear margins) provided 0% rates of recurrence in the reported cases. Due to the rarity of these tumors and recent histological reclassification, a comprehensive review of this condition will assist clinicians in their management of it.

## 1. Introduction

Myxomas are a group of uncommon benign tumors of mesenchymal origin that are locally invasive and often have high rates of recurrence. Rarely, myxomas involve the head and neck region, accounting for 0.1% of all head and neck tumors and less than 0.5% of sinus and nasal cavity tumors. Of these tumors, 75% occur in the mandible and maxilla [1].

Sinonasal myxomas (SNMs) are extremely rare, and the exact etiology and pathogenesis are unclear. These lesions are often misdiagnosed as odontogenic myxomas or odontogenic fibromyxomas. However, there appears to be a clear preponderance for SNMs to occur in young children and they occur almost exclusively in the maxilla and paranasal sinuses. This is in contrast to odontogenic myomas (OMs), which occur most commonly in the mandible followed by the maxilla, and in a wider age group [2]. Recently, infantile sinonasal tract myxomas have been identified as a distinct entity by the World Health Organization (WHO) within the spectrum of tumors driven by the WNT/β-catenin pathway [3].

To our knowledge, we report the largest literature review of the topic to date, including two new cases of sinonasal myxoma with orbital involvement. We have comprehensively reviewed the literature to provide summarized clinical characteristics of this lesion to aid the clinician in recognizing and diagnosing infantile sinonasal myxoma.

## 2. Materials and Methods

A retrospective review of published data (between 1945 and 2023) on sinonasal myxoma was conducted. Data were collected from each of the identified articles on the age of presentation, sex, initial presentation, tumor location, imaging, pathology, and treatment. Medline, PubMed, and Google Scholar were searched for case reports and reviews in the English language using keywords—*sinonasal myxoma*, *orbit*, *maxillary sinus*, *sinus*, *sinuses*—which were screened for relevance and included if a case was described with a patient age < 3. The cases identified are summarized and reported in Table S1.

## 3. Results

### 3.1. Case 1

A thirteen-month-old male presented with a four-week history of a rapidly enlarging mass of the left lateral nose and associated epiphora. Clinical examination revealed left facial erythema with associated bruising and left nasal obstruction. The baseline ophthalmological assessment was normal. Clinical examination was otherwise unremarkable.

**Figure 1:**
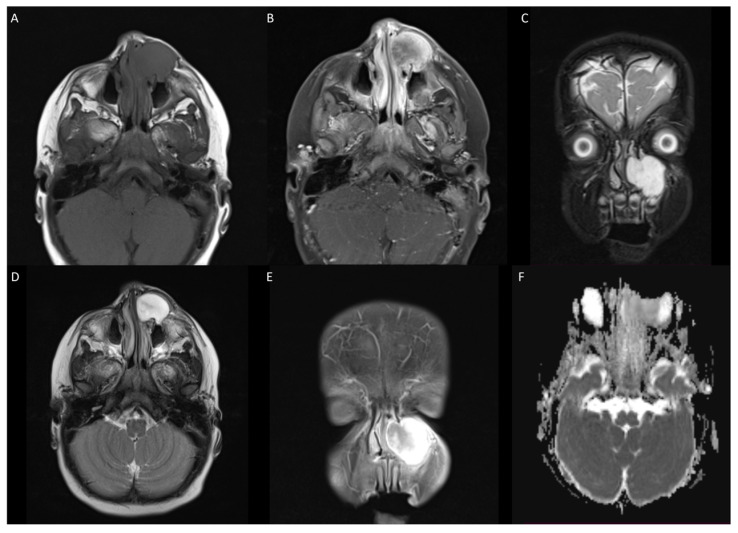
(**A**) T1 weighted image showing hypointense lesion in left maxilla extending infraorbitally. (**B**,**E**) T1 with contrast showing avid heterogenous enhancement of lesion. (**C**) T2 coronal demonstrating superior and inferior extension of lesion. (**D**) T2 fat-suppressed axial with hyperintense lesion. (**F**) DWI showing no apparent hyperintensity or low ADC values.

Magnetic resonance imaging (MRI) of the head confirmed a left-sided mass over the frontal process of the maxilla measuring 22 × 25 × 25 mm (13.75 cm^3^), with extension into the inferomedial aspect of the left orbit, through the anterior wall of the maxillary sinus and into the left nasal cavity. The appearance of the globes, optic nerve, and extraocular muscles was normal. The lesion was T1 hypointense with avid heterogenous contrast enhancement and T2 hyperintense (Figure 1).

**Figure 2:**
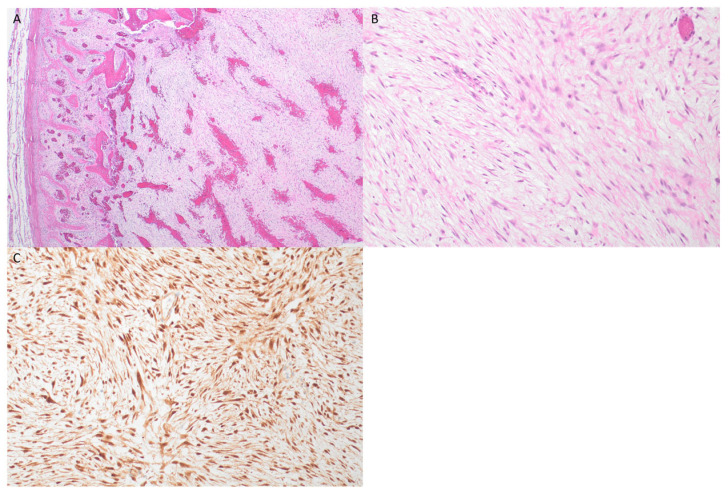
(**A**) H&E ×4—a low power image showing loose myxoid stroma with spindle cells, and a peripheral rim of reactive new bone formation which was present around portions of the lesion. (**B**) H&E ×20—a higher power image demonstrating bland spindle and stellate cells with a tissue-culture-like appearance similar to that seen in fasciitis-like lesions. (**C**) Beta-catenin immunohistochemistry (from the initial biopsy sample) confirming diffuse nuclear expression (with weak cytoplasmic staining), indicating alteration of the WNT-pathway.

Biopsy of the maxillary sinus mass showed features of a fasciitis-like tumor with a provisional diagnosis of “cranial fasciitis-like lesion with WNT/β-catenin dysregulation” [4,5]. Immunohistochemistry was negative for smooth muscle actin, desmin, and myogenic markers (myogenin and MyoD1), but demonstrated diffuse positive nuclear staining for ß-catenin (Figure 2), which is supportive of SNM with WNT/β-catenin pathway dysregulation.

The patient underwent further excision/debulking of the tumor. Intraoperatively, it was noted that there was a large gelatinous fungating mass with bony invasion into the nasal wall and inferonasal orbit. The histology of the resected lesion was the same as the biopsy and showed a low to medium cellularity proliferation of spindle cells in a myxoid stroma, with much of the lesion having a “tissue-culture” like appearance. Mitotic activity to 2 mitoses/10 hpf was noted, with no atypical forms, and there was no significant cytological atypia or necrosis. A rim of reactive new bone with tumor involvement was seen at the periphery of the lesion on histological examination. Bone margins were resected, and soft tissue margins anteriorly were achieved operatively; however, on microscopic examination, the tumor was seen to transect the surgical resection plane over a broad front and was pathologically confirmed as incompletely excised.

At the time of resection, tumor samples were sent for PCR testing of the *CTNNB1* gene, which was not identified; however, it is noted that this testing was confined to exon 3 (codons 41 and 45), which includes the hotspot mutations typically seen in desmoid fibromatosis tumors only. Testing of the *APC* gene was not performed at that time although the possibility of underlying germline mutation was noted.

**Figure 3:**
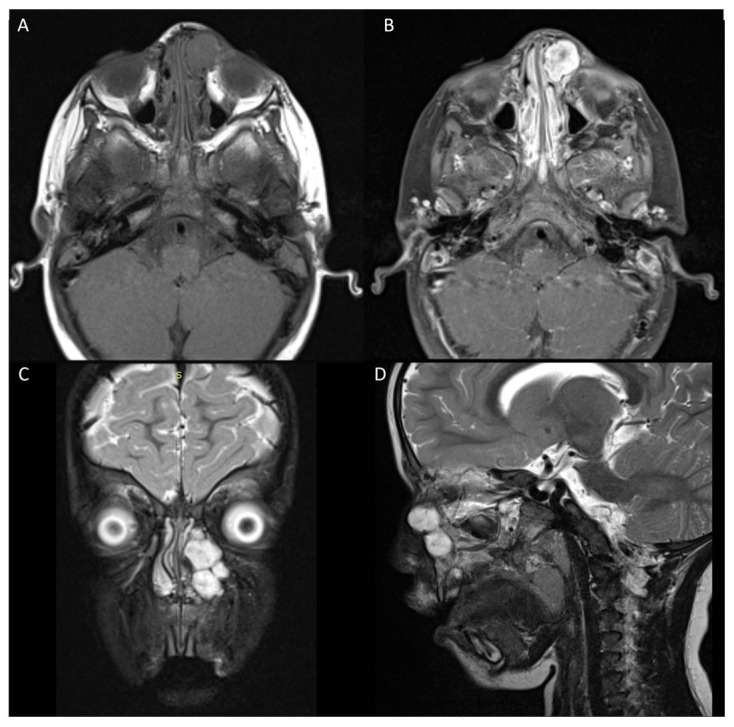
Recurrence of tumor. (**A**) T1 weighted hypointense lesion in location of previously excised tumor. (**B**) T1 heterogenous contrast enhancement consistent with previous tumor. (**C**,**D**) Coronal STIR and T2 sagittal showing hyperintensity consistent with recurrence.

Six months post-surgery, the patient presented with a two-week history of an enlarging firm mass in the left medial canthal area with epiphora. An MRI scan showed a likely recurrence of lesion 32 × 16 × 17 mm (8.70 cm^3^) in the left maxillary sinus invading into the left ethmoid and inferomedial left orbit (Figure 3). A secondary excision was performed which involved more extensive resection of the involved bone and soft tissue.

Histopathology confirmed tumor involvement in the bony margin. Although further bone was removed (drilled) at the time of surgery and there was no clinical or radiological evidence of recurrence, adjuvant chemotherapy was recommended. He was placed on vinblastine and methotrexate with good results. At the time of writing, the patient has been followed up and is disease-free at five years of age.

### 3.2. Case 2

A fourteen-month-old female presented with a seven-week history of slowly enlarging left cheek mass. Clinical examination showed left hyperglobus, proptosis, and significant conjunctival injection. Cycloplegic refraction right eye +1.0 and left eye +0.5/+4.00 × 60 demonstrated a high degree of oblique astigmatism in the left eye. Fundoscopy was unremarkable with normal optic nerve parameters. Sinonasal examination showed complete obstruction of the left nasal passage and partial obstruction of the right. The large firm mass was present over her maxilla and palpable through the gingival buccal sulcus.

**Figure 4:**
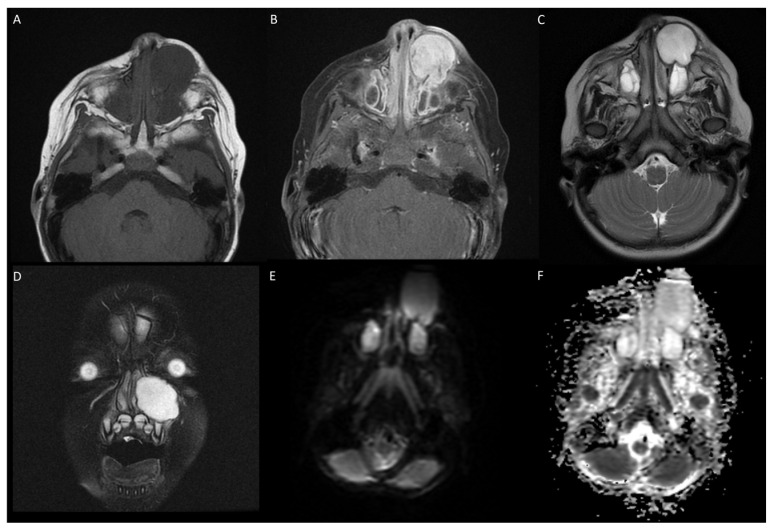
(**A**) T1 weighted image showing hypointense lesion in left maxillary region extending into infraorbital space. (**B**) T1 contrast showing avid enhancement of lesion. (**C**) T2 weighted axial showing homogenous hyperintense lesion. (**D**) T2 fat-suppressed coronal. (**E**,**F**) DWI and ADC showing no abnormal diffusion restriction.

A magnetic resonance imaging (MRI) scan showed a large mass of 32 × 26 × 29 mm (24.13 cm^3^) arising from the left maxilla extending to the inferior orbit (Figure 4). There was obstruction of the left nasal passage with a significant deviation of the nasal septum. The lesion demonstrated T1 hypointensity, T2 hyperintensity, and avid heterogenous contrast enhancement. There was nil or minimal diffusion restriction on ADC images. Whole Body FDG PET/CT demonstrated a heterogeneous uptake in the left maxillary lesion with central photopenia. The SUVmax was 2.7; there was destruction of the inferior orbital wall with preservation of the fat pad.

Following the initial biopsy diagnosis, the patient underwent surgical excision via a combined open and endoscopic approach with stenting of the lacrimal system and rib graft reconstruction. Intraoperatively, there was a large mass in the gingivobuccal groove with erosion into the lateral nasal wall and inferior orbital rim. There was bony destruction of the alveolar and zygomatic processes of the maxilla and orbital floor.

**Figure 5:**
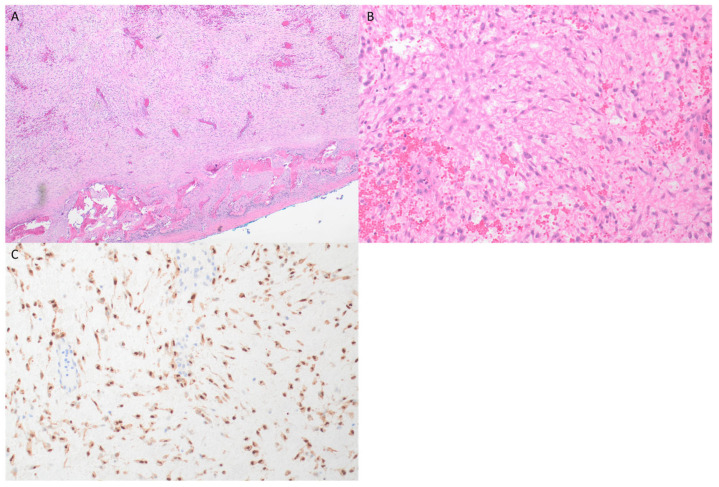
(**A**) H&E ×4—low power image also showing loose myxoid stroma, although less prominent than with case 1; peripheral rim of reactive new bone was also seen focally around lesion. (**B**) H&E ×20—higher power showing spindle, stellate and some larger, more epithelioid cells, although without significant cytological atypia. (**C**) Beta-catenin ×20 demonstrates diffuse nuclear expression of tumor cells, noting that vessels within lesion are not stained.

Histological examination showed mesenchymal cells set on a myxoid background with round to spindle cells, focally forming short fascicles. Immunohistochemistry showed patchy expression for smooth muscle actin, with negative myogenic markers, and diffuse positive nuclear expression for ß-catenin (Figure 5). Chromosome microarray was undertaken on this tumor, which identified biallelic *APC* gene loss. Germline testing for *APC* deletion was not performed. Targeted next-generation RNA sequencing on tumor tissue did not find any *USP6* gene fusion or *CTNNB1* mutation. The patient is disease free at twelve-month follow-up, both clinically and radiologically.

### 3.3. Literature Review

A systematic search of PUDMED, MEDLINE, and Google Scholar data was conducted to find articles describing infantile sinonasal tract myxoma. A total of forty-eight patients were identified in the literature. Results are summarized in Table S1.

The average age of diagnosis of infantile sinonasal myxoma was 16.6 months. Of the forty-eight infantile cases, twenty-five were in males (52.1%), eighteen were in females (37.5%), and five had no sex listed (10.4%), a ratio of 1:1.39 F:M.

The most common presenting symptom was localized swelling or enlarging mass in the nasal and maxillary sinus region (96%). Other less commonly reported symptoms included nasal obstruction in four cases (8.3%), epistaxis in one case (2.1%), and fever in one case (2.1%). The reported cases of SNM most commonly involve the maxillary sinus (83.3%) [1,2,6,7,8,9,10,11,12,13,14,15,16,17,18,19,20,21,22,23,24,25,26,27,28,29,30,31] and much more rarely the orbit (22.9%) [11,12,14,21,23,27,30,32,33]. Only ten cases involved the nasal cavity and other sinuses (20.8%) [2,12,21,24,33].

Imaging modalities reported in the literature include three early cases that use X-ray only (6.25%), thirty-four cases with CT scan results (70.8%), fifteen cases with MRI (31.3%), and one case that utilized ultrasound (2.1%).

The outcomes of the cases reported in the literature vary significantly depending on the treatment. Of the forty-eight cases reported in the literature, thirty-nine (81.25%) had follow-up data recorded. Five cases demonstrated recurrence (10.4%), with an average time to recurrence of 8.8 months and an average follow-up duration of 46.9 months.

Treatment data were available for forty-seven of the forty-eight cases. Eight underwent partial resection or debulking (16.7%) and thirty-nine gross surgical excision (81.3%), with clear margins confirmed in twenty-five of these cases. Partial resection or complete resection without demonstration of clear margins occurred in eight and fourteen cases, respectively. There were five cases of reported recurrence; three occurred in patients who underwent partial resection and two in patients with complete resection without confirmation of clear margins. In cases with orbital involvement, complete resection with clear margins was achieved in four cases and without confirmation of clear margins in seven cases. Importantly, there were no recurrences in patients with confirmed clear margins.

## 4. Discussion

The term “myxoma” is a descriptive one, used to describe a mesenchymal tumor with a prominent stromal mucopolysaccharide matrix of sulphated and non-sulfated glycosaminoglycans. However, not all “myxomas” are the same, with those arising in different sites generally frequently representing distinct entities with distinct pathogenesis and molecular features. Head and neck myxomas were first described by Virchow in 1871 and most commonly occur in the mandible and maxilla [34]. Head and neck myxomas account for as little as 0.1% of all head and neck tumors [1]. Myxomas of the paranasal sinus and nasal cavity are extremely rare and sinonasal myxomas have previously eluded pathological classification by the WHO as a separate entity and have often been misreported in the literature as “odontogenic myxomas”. However, SNM has relatively recently been appreciated as a distinctive clinicopathological entity predominantly arising from the maxillary bone, first included as a specific entity in the WHO Classification of Tumor Pathology only in the recently published Pediatric Tumor Classification [35].

Orbital involvement in infantile sinonasal tract myxomas is reported in approximately 20% of cases [11,12,14,21,23,27,30,32,33,36,37]. Due to the rarity of sinonasal myxoma with orbital involvement in infants, we have performed a literature review of the clinical presentation, imaging findings, histology, genetics, and long-term outcomes (Table S1).

### 4.1. Demographics

Odontogenic and non-odontogenic myxomas of the head and neck occur most commonly in the 2nd to 4th decades of life but have been reported from 10 to 61 years of life [38]. As sinonasal myxomas were not recognized until recently as a separate lesion, they were often misreported in the literature as odontogenic myxomas. The majority of odontogenic myxomas arise in the mandible, unlike SNM, which is predominantly a maxillary lesion. There is also a distinct predilection for these lesions to affect males (1:1.39, F:M).

### 4.2. Clinical Presentation and Imaging

Infantile sinonasal myxomas are generally slow-growing, expansile, and locally destructive; they are infiltrative lesions with the potential for locally aggressive behavior due to their ability to erode bone. These lesions most frequently present as painless swelling in the nasal and paranasal regions. Of the forty-eight reported cases of infantile sinonasal myxoma, all arose from either the maxilla or the sinonasal complex, with forty described as involving the maxilla and maxillary sinuses (83.3%) [1,2,6,7,8,9,10,11,12,13,14,15,16,17,18,19,20,21,22,23,24,25,26,27,28,29,30,31], eleven cases involving the orbit (22.9%) [11,12,14,21,23,27,30,32,33], and ten involving the nasal cavity/paranasal sinuses (20.8%) [2,12,21,24,33,39,40,41,42,43]. Recently, maxilla or maxillary sinus involvement was reported in 95–100% of cases of sinonasal myxomas [3]. Both of our reported cases have orbital involvement, adding to only eleven other reported cases.

CT imaging shows homogenous solid expansile masses with well-defined borders and heterogenous contrast enhancement. The MRI of sinonasal myxoma demonstrates a T1 hypointense lesion with variable contrast enhancement and T2 hyperintensity with incomplete fluid suppression on T2 FLAIR. Typically, these lesions do not show diffusion restriction, supporting general low cellularity.

### 4.3. Histopathology and Immunohistochemistry

Macroscopic examination of SNM reveals glossy, gelatinous-to-firm-consistency, pink/gray masses with irregular borders that are not grossly encapsulated, or may possess a pseudocapsule, and that frequently show a rim of reactive bone. Histology typically shows a loose, relatively hypocellular proliferation of either stellate, spindle, or round cells within a myxoid stroma. Tumor cells may have a “tissue-culture”-like quality, showing morphological overlap with fasciitis-like lesions (including cranial fasciitis in young children). Elongated fascicles of tumor cells as seen in desmoid fibromatosis are not a feature. Tumor necrosis and odontogenic epithelium are absent, the latter by definition, as the presence of odontogenic epithelium distinguishes these lesions from odontogenic myxoma. Mitotic figures may be seen in generally low numbers without atypical forms, and pleomorphism of tumor cells is not a major feature [18,20,27,44].

Immunohistochemistry may show variable smooth muscle actin expression (indicating myofibroblastic differentiation), although this is not always seen. Myogenic markers (desmin, myogenin, MyoD1) are negative, excluding the differential diagnostic consideration of embryonal rhabdomyosarcoma, which frequently also has myxoid stroma, and shows locally aggressive features on imaging. Importantly, all lesions described to date have shown diffuse aberrant nuclear expression for ß-Catenin indicating dysregulation of the WNT/ß-Catenin pathway, representing the most useful immunohistochemistry stain to confirm the diagnosis. In contrast, odontogenic myxomas do not show nuclear ß-Catenin expression.

### 4.4. Molecular Pathology

Virtually all tumors with reported molecular results have identified either gain of function mutations of the CTNNB1 gene, representing the majority of cases, or biallelic adenomatous polyposis coli (APC) gene inactivation, with both resulting in activation of the WNT/ß-Catenin pathway [3,45]. Of note, the activating CTNNB1 gene mutations are mostly located in the ubiquitination recognition motif of the CTNNB1 gene, which is different to that of desmoid fibromatosis (typically involving exon 3, encoding the phosphorylation domain of CTNNB1), although occasional SNM cases have also reported exon 3 CTNNB1 mutation [3]. Cases with biallelic APC gene inactivation include occasional cases with confirmed germline APC gene mutation, suggesting that at least a subset may be associated with underlying familial adenomatous polyposis [46].

### 4.5. Treatment

Surgical excision is the treatment of choice for sinonasal myxoma. There are no clear surgical guidelines for the management of infantile SNM [10]. Due to the invasive and destructive nature of sinonasal myxoma and their lack of encapsulation, these lesions are at risk of recurrence, predominantly when incompletely excised. The role of radiotherapy and systemic chemotherapy in the treatment of these lesions is not well documented. In one of our cases, we used chemotherapy due to the recurrence of the mass to good effect.

## 5. Conclusions

Infantile SNM is an uncommon entity, with only small numbers of cases with orbital involvement described in the literature, such that these can present a challenging diagnostic dilemma. SNM is emerging as a distinct WNT/β-catenin pathway-driven tumor with increasingly well-defined clinical, demographic, and molecular genetic findings, and characteristic histopathology and immunohistochemical features within the clinical context. No clear guidelines for management of this disorder exist; however, complete surgical excision with pathological confirmation of clear margins results in the lowest rates of recurrence. One of our cases was particularly challenging, with multiple recurrences, and was successfully treated with chemotherapy. Further research into this area may provide targeted therapies and clearer diagnostic guidelines, especially when primary surgical excision is not possible.

## Supplementary Materials

The following supporting information can be downloaded at: https://www.mdpi.com/article/10.3390/jcm13226818/s1, Table S1: A summary of the literature—sinonasal myxoma.
