# Supplementary material for: Infantile Sinonasal Tract Myxomas with Orbital Involvement: Presentation of Two Cases and Comprehensive Literature Review
Source: J Clin Med. 2024 Nov 13;13(22):6818. doi: 10.3390/jcm13226818 (PMC11595077; doi:10.3390/jcm13226818)
Supplement: Supplementary file 1 [file jcm-13-06818-s001.zip › jcm-3295984-supplementary.pdf]

Table S1. Summary of the Literature – Sinonasal Myxoma.

Author/Date	Site	Age (months)	Sex	Presenting symptom	Imaging	Treatment	Follow up outcomes	Follow up
Burns et al. 2024	Maxillary sinus + orbit	14	F	Slowly enlarging mass with proptosis	MRI & PET	Surgical Excision	Disease free at follow up	36
Burns et al. 2024	Maxillary sinus + orbit	13	M	Rapidly enlarging painless mass, epiphora	MRI & CT	Surgical Excision, Chemotherapy	Recurrence at 6 months and 13 months	18
Greenfield, 1951 [26]	Maxillary sinus	24	M	Painless, Facial swelling	Xray			
Canalis, 1976 [24]	Maxilla	24	M	Painless, Facial swelling	Xray	Surgical Excision	Disease free at follow up	36
Yao-Shi Fu, 1977 [25]	Maxillary sinus	15	F	Nasal obstruction	Xray	Surgical Excision	Disease free at follow up	108
Harris, 1977 [27]	Maxilla	12	F	Mass		Surgical Excision	Disease free at follow up	12
Smith, 1977 [36]	Maxilla	15	F	Facial swelling		Surgical Excision	Recurrence	96
James, 1987 [30]	Maxilla	11	F	Pyrexia, Facial swelling,	CT	Chemotherapy, Surgical Excision	Disease free at follow up	96
Leiberman, 1990 [13]	Maxilla	18	F	Facial swelling	CT	Biopsy, Surgical Excision	Disease free at follow up	36
	Maxilla + orbit	15	M	Facial swelling	CT	Biopsy, Surgical Excision	Disease free at follow up	
Hayes, 1991 [28]	Maxillary sinus	12	F	Facial swelling	CT & MRI	Fina needle aspiration		
Ang, 1993 [20]	Maxillary & ethmoid sinus	13	M	Facial swelling	CT	Surgical excision	Disease free at follow up	10
		14	F	Mass		Surgical excision	Recurrence at 5 months, re-excision No disease at subsequent follow up	84
Heffner, 1993 [29]	Maxillary sinus	24	M	Facial swelling		Biopsy, Surgical excision	Disease free at follow up	168
	Maxillary sinus	12	M	Facial swelling	CT	Biopsy, Surgical excision		
Caleffi, 1994 [23]	Maxillary sinus	24	M	Painless, Facial swelling	CT	Surgical excision	Disease free at follow up	12

Brewis, 2000 [22]	Maxillary sinus	13	M	Painless, Facial swelling	CT	Biopsy, Surgical excision	Disease free at follow up	4
Fenton, 2003 [18]	Nasal cavity & Maxillary sinus + orbit	17	M	Enlarging mass	CT	Surgical Excision	Disease free at follow up	16
Wachter, 2003 [17]	Maxillary sinus	13	M	Enlarging mass	CT & MRI	Biopsy, Surgical excision	Disease free at follow up	24
	Maxilla + orbit	19	F	Enlarging mass	CT	Biopsy, Surgical excision	Disease free at follow up	24
Rotenberg, 2004 [16]	Maxilla	13	F	Enlarging mass	CT	Biopsy, Surgical excision	Disease free at follow up	48
	Maxilla + orbit	18	F	Enlarging mass	CT	Biopsy, Surgical excision	Disease free at follow up	168
	Maxilla + orbit	16	M	Facial swelling	CT	Biopsy, Surgical excision	Disease free at follow up	84
Prasannan, 2005 [34]	Maxillary sinus	20	F	Facial swelling	CT & MRI	Biopsy, Surgical excision	Disease free at follow up	8
Boussault, 2006 [21]	Maxillary sinus	14	M	Facial swelling, Enlarging mass	CT & MRI	Biopsy, Surgical excision		
Odman, 2006 [33]	Maxillary Sinus & Ethmoid	21	M	Painless, Mass	CT & MRI	Surgical Resection	Recurrence requiring surgical excision, 2 years after excision disease free	12
King, 2008 [32]	Maxilla	18	M	Facial swelling	CT	Biopsy, enucleation	Disease free at follow up	18
		17	M	Enlarging mass	CT	Biopsy, Surgical excision	Disease free at follow up	24
Iatrou, 2010 [39]	Sinonasal tract	12	M	Facial swelling	MRI	Biopsy, Surgical excision	Disease free at follow up	42
Safadi, 2011 [43]	Sinonasal myxoma	20	M	Enlarging mass	CT	Biopsy, surgical excision	Disease free at follow up	12
Jiang, 2012 [40]	Sinonasal	20	M	enlarging mass	CT & MRI	Surgical excision	Disease free at follow up	12
Kansy, 2012 [11]	Maxillary sinus + orbit	12		Painless, swelling	CT & MRI	Surgical excision	Disease free at follow up	24

	Maxillary sinus, Nasal passage + orbit	11		Epistaxis, painful, facial swelling	Ultrasound & MRI	Surgical excision	Disease free at follow up	96
Rios, 2012 [35]	Maxillary sinus	11	F	Painless, facial swelling	CT	Biopsy, Surgical excision	Disease free at follow up	48
Chen, 2013 [8]	Maxillary sinus + orbit	15	M	Facial swelling, Nasal obstruction	CT	Surgical excision	Disease free at follow up	27
Kadlub, 2014 [31]	Maxilla	18	F	Facial swelling	CT	Enucleation of the mass	Disease free at follow up	72
	Maxilla	23	F	Facial swelling	CT	Enucleation of the mass	Disease free at follow up	48
	Maxilla	21	F	Facial swelling	CT	Enucleation of the mass	Recurrence	18
	Maxilla	14	M	Facial swelling	CT	Enucleation of the mass	Recurrence	12
Zainine, 2014 [38]	Maxilla	3	F	Nasal obstruction, Nasal discharge	CT & MRI	Biopsy, Surgical excision	Disease free at follow up	30
Kelly, 2015 [41]	Nasal cavity	5	M	Facial swelling	CT & MRI	Biopsy, Debulking, Surgical excision		
Hansen, 2016 [10]	Orbit	36	M	Facial swelling		Surgical excision		
Subramaiam, 2016 [37]	Maxilla	24		Painless, Facial swelling		Biopsy, Surgical excision	Disease free at follow up	103
	Maxilla	24		Facial swelling		Biopsy, Surgical excision	Disease free at follow up	44
	Maxilla	24		Facial swelling		Biopsy, enucleation	Disease free at follow up	10
Yucel Ekici, 2018 [1]		0 (1 day)	F	Large Mass	MRI	Surgical excision	Disease free at follow up	48
Gandhi, 2019 [9]	Maxilla + orbit	36	F	Painful, Facial swelling	CT	Biopsy, Surgical excision		
Mewar, 2020 [2]	Maxilla + paranasal sinus	11	M	Facial swelling	CT & MRI	Biopsy, surgical excision	Disease free at follow up	48
Kondamuri, 2021 [12]	Sinonasal + orbit	9	M	Facial swelling, Nasal obstruction	CT & MRI	Surgical excision		
Phillips, 2021 [42]	Nasal mass	15	M	Enlarging mass	CT & MRI	Surgical excision		
